# Precipitation Chemistry and Occurrence of Acid Rain over the Oil-Producing Niger Delta Region of Nigeria

**DOI:** 10.1100/tsw.2010.61

**Published:** 2010-04-01

**Authors:** John Kanayo Chukwu Nduka, Orish Ebere Orisakwe

**Affiliations:** ^1^ Pure and Industrial Chemistry Department, Nnamdi Azikiwe University, Awka, Anambra State, Nigeria; ^2^ Toxicology Unit Department of Clinical Pharmacy, Faculty of Pharmacy, University of Port Harcourt, Rivers State, Nigeria

**Keywords:** oil exploration, chemical parameters, environmental pollution, degradation, health problem

## Abstract

This study investigated the nitrate, sulfate, total dissolved solid (TDS), electrical conductivity, total hardness (TH), and bicarbonates of rainwater samples collected from Warri and Port Harcourt between April–June, July–August, and September–October of 2005 and 2006 to depict onset of rainy season, mid-rainy season, and end of rainy season for the two major crude oil–producing cities of the Niger Delta region of Nigeria (although Port Harcourt is also noted for non-oil manufacturing industries). The same was done in Awka, a non-oil producing city in the hinterland of southeastern Nigeria. In each of the cities, rain samples were collected from three points in a triangular equilibrium using a clean plastic basin fastened to a table 2 m above ground level and 115 m away from tall buildings and trees. The parameters were determined after filtering, using their respective standard methods. Averages of 1.50, 1.81, 1.13 and 2.14, 1.50, 1.86 mg/l of nitrate for April–June, July–August, and September–October were recorded for Warri in 2005 and 2006, respectively. While 15.21, 3.23, 22.31 and 20.89, 9.96, and 14.27 mg/l were recorded in Port Harcourt. Sulfate levels for Warri and Port Harcourt for the same periods are follows: 1.38, 1.88, 1.06, 1.50, 1.43, 1.50 and 2.64, 1.15, 5.88, 4.73, 1.90, 1.55 mg/l, respectively. Nitrate levels were higher than sulfate. Other parameters include TDS (5.44, 4.79, 3.30 and 7.63, 3.69, 2.56 mg/l for Warri in 2005 and 2006; 12.57, 2.07, 25.214 and 28.87, 6.73, 7.80 mg/l for Port Harcourt for the same periods). Other parameters also varied in that order for the 2 years in same cities. Crude oil exploration and gas flaring in the Niger Delta, and multiplicity of cottage industries in Awka, impacted on the inorganic ion pollution of the rainwater. This may have public health implications in the region.

## INTRODUCTION

The Niger Delta region of Nigeria is regarded as the treasure bed of the country as more than 95% of Nigeria's foreign earnings come from there, and it is characterized by an interwoven network of ditches, ponds, rivulets, streams, lakes, rivers, estuaries, and a characteristic shallow water table that, incidentally, is the only source of domestic water supply. Oil exploration and exploitation have been dominant activities in the area for more than 40 years. Crude and refined oil spillages, gas flares, and industrial effluents have impacted negatively on the arable land and the aquatic ecosystem, and there is serious pollutant-atmospheric interaction.

Streams, shallow wells, and boreholes form the major sources of potable water supply for the different communities in the Niger Delta. Rainwater is the major alternative source of water for drinking, cooking, laundry, and sundry domestic needs. Water treatment is rarely carried out at the household level, while adequate and safe drinking water provision is the federal and state governments' nonpriority event. Our previous studies showed that the pH of rainwater in the Niger Delta cities of Warri and Port Harcourt in 2005 and 2006 were acidic[1].

To ascertain the suitability of rainwater for drinking, cooking, laundry, and sundry domestic needs, the present work investigated the physicochemical properties of rainwater in the Niger Delta cities of Warri and Port Harcourt. The data are compared with Awka, another city outside the Niger Delta, where there is no crude oil exploration activity. The data will not only be a useful guide for public health workers and community-based physicians, but they will also serve as a baseline against which future anthropogenic and other effects may be assessed.

## MATERIALS AND METHODS

Rainwater samples were collected from Warri and Port Harcourt, two cities of the main oil- and gas-rich region of Nigeria's Niger Delta. (Port Harcourt is also noted for non-oil manufacturing industries.) Awka, the capital of Anambra State, is 255 and 205 km from Port Harcourt and Warri, respectively, but is 42 and 58 km from Nnewi and Onitsha (two industrialized cities in close proximity to Awka with obvious environmental effect to the latter), respectively. It is outside the crude oil–exploring area of the Niger Delta. The rainwater was sampled between April–October 2005 and 2006; namely, April–June 2005, July–August 2005, September–October 2005, April–June 2006, July–August 2006, and September–October 2006. In each city, the samples were collected from three points in a triangular equilibrium using a clean plastic basin fastened to a table 2 m above ground level and 115 m away from tall buildings and trees.

The samples were filtered using filter paper with a pore size of 5 µm before analysis. Nitrate was analyzed using the Bruccine colorimetric method of the Association of Official Analytical Chemists[2]. Sulfate was determined by the titrimetic method using EDTA[3]. A digital conductometer (Consort k 120, Belgium) was used to determine electrical conductivity after standardizing the rainwater samples in a beaker with 0.1 *M* KCl. Total dissolved solid (TDS) was determined by measuring a portion of the water samples and filtering through a standard glass fiber filter with a pore size of 5 µm, and the filtrate evaporated to dryness in a weighed dish and dried to constant weight at 180$^{o}$C. The increase in dish weight represents the TDS[3]. The complexiometric titration method was employed in the determination of total hardness (TH) of the water samples, while bicarbonate was determined by electrometric titration[3].

## RESULTS

Tables 1 and 2 show the physicochemical parameters of rainwater in Warri between April–October 2005 and April–October 2006, respectively. There was significant periodic variation in the level of hardness and nitrate of rainwater in 2005. There were also significant periodic variations in TH, TDS, and electrical conductivity of the rainwater in 2006. In all, the physicochemical parameters of Warri were found to be significantly different from Awka rainwater samples for both 2005 and 2006.

**TABLE 1**
**Physicochemical Parameters of Rainwater in Warri between April–October 2005**

| Period | TH (mg/l) | $NO^-_3$ (mg/l) | $HCO^-_3$ (mg/l) | $SO_4^{2-}$ (mg/l) | TDS (mg/l) | Electrical Conductivity (µ.S/cm) |
|---|---|---|---|---|---|---|
| April–June 2005 | 49.08 ± 0.003 (61.14 ± 0.003) | 1.50 ± 0.007 (17.76 ± 0.322) | 0.99 ± 0.025 (1.12 ± 0.063) | 1.38 ± 0.011 (1.88 ± 0.012) | 5.44 ± 0.040 (4.79 ± 0.006) | 10.30 ± 0.051 (10.41 ± 0.009) |
| July–Aug 2005 | 47.83 ± 0.281 (59.51 ± 0.006) | 1.81 ± 0.006 (23.31 ± 0.000) | 0.85 ± 0.003 (0.63 ± 0.000) | 1.88 ± 0.011 (0.95 ± 0.000) | 4.79 ± 0.007 (6.88 ± 0.013) | 9.50 ± 0.004 (14.44 ± 0.003) |
| Sept–Oct 2005 | 62.21 ± 0.051 (58.01 ± 0.003) | 1.13 ± 0.011 (23.16 ± 0.003) | 0.82 ± 0.008 (0.90 ± 0.085) | 1.06 ± 0.006 (2.20 ± 0.003) | 3.30 ± 0.004 (6.56 ± 0.028) | 3.86 ± 0.011 (14.35 ± 0.000) |

Data presented as mean ± SEM.

Values in parentheses represent data from Awka City.

N = 8–15 corresponding to episodes of rainfall within the stipulated period.

**TABLE 2**
**Physicochemical Parameters of Rainwater in Warri between April–October 2006**

| Period | TH (mg/l) | $NO^-_3$ (mg/l) | $HCO^-_3$ (mg/l) | $SO_4^{2-}$ (mg/l) | TDS (mg/l) | Electrical Conductivity (µ.S/cm) |
|---|---|---|---|---|---|---|
| April–June 2006 | 49.59 ± 0.002 (60.51 ± 0.006) | 2.14 ± 0.006 (11.13 ± 0.003) | 0.69 ± 0.114 (0.67 ± 0.004) | 1.50 ± 0.000 (2.26 ± 0.006) | 7.63 ± 0.010 (5.65 ± 0.000) | 15.00 ± 0.058 (11.84 ± 0.006) |
| July–Aug 2006 | 47.98 ± 0.131 (58.77 ± 0.003) | 1.50 ± 0.000 (8.91 ± 0.175) | 0.86 ± 0.003 (0.53 ± 0.000) | 1.43 ± 0.003 (2.25 ± 0.126) | 3.69 ± 0.004 (4.80 ± 0.000) | 7.43 ± 0.006 (6.96 ± 0.009) |
| Sept–Oct 2006 | 47.16 ± 0.022 (60.14 ± 0.016) | 1.86 ± 0.029 (6.60 ± 0.013) | 0.92± 0.001 (0.63 ± 0.006) | 1.50 ± 0.000 (1.71 ± 0.006) | 2.56 ± 0.239 (5.000 ± 0.000) | 5.02 ± 0.118 (8.35 ± 0.063) |

Data presented as mean ± SEM.

Values in parentheses represent data from Awka City.

N = 8–15 corresponding to episodes of rainfall within the stipulated period.

The physicochemical parameters of rainwater in Port Harcourt between April–October 2005 and April–October 2006 are shown on Tables 3 and 4, respectively. There were low nitrate, TH, TDS, and electrical conductivity levels in July–August of 2005. These values also showed significant periodic variation in 2005. The physicochemical parameters significantly varied from the Awka data. Nitrate, TDS, and electrical conductivity were lower in July–August 2006, whereas nitrate was highest within the same period. With the exception of bicarbonates, all the parameters showed significant variations when compared with the Awka data.

## DISCUSSION

Nigerians depend heavily on rainwater during the rainy season (April–October) for their water supply; hence the need to assess its suitability for drinking, cooking, and other domestic uses. The present study has investigated the suitability of rainwater as an alternative source of potable water supply in the Niger Delta. This may be one of the few documented studies on precipitation chemistry and occurrence of acid rain over the oil-producing Niger Delta area of Nigeria. Particulates, gases, and aerosols in the atmosphere are

**TABLE 3**
**Physicochemical Parameters of Rainwater in Port Harcourt between April–October 2005**

| Period | TH (mg/l) | $NO^-_3$ (mg/l) | $HCO^-_3$ (mg/l) | $SO_4^{2-}$ (mg/l) | TDS (mg/l) | Electrical Conductivity (µ.S/cm) |
|---|---|---|---|---|---|---|
| April–June 2005 | 47.67 ± 0.021 (61.14 ± 0.003) | 15.21± 0.064 (17.76 ± 0.322) | 0.60 ± 0.006 (1.12 ± 0.006) | 2.64 ± 0.002 (1.88 ± 0.012) | 12.57 ± 0.008 (4.79 ± 0.006) | 25.87 ± 0.323 (10.41 ± 0.009) |
| July–Aug 2005 | 49.52 ± 0.070 (59.51 ± 0.006) | 3.23 ± 0.005 (23.31 ± 0.000) | 0.86 ± 0.019 (0.63 ± 0.000) | 1.15 ± 0.554 (0.95 ± 0.000) | 2.07 ± 0.003 (6.88 ± 0.013) | 4.23 ± 0.051 (14.44 ± 0.003) |
| Sept–Oct 2005 | 49.59 ± 0.173 (58.01 ± 0.003) | 22.31 ± 0.038 (23.16 ± 0.003) | 0.69 ± 0.184 (0.90 ± 0.085) | 5.88 ± 0.043 (2.20 ± 0.003) | 25.214 ± 0.009 (6.56 ± 0.028) | 51.214 ± 0.006 (14.35 ± 0.000) |

Data presented as mean ± SEM.

Values in parentheses represent data from Awka City.

N = 8–15 corresponding to episodes of rainfall within the stipulated period.

**TABLE 4**
**Physicochemical Parameters of Rainwater in Port Harcourt between April–October 2006**

| Period | TH (mg/l) | $NO^-_3$ (mg/l) | $HCO^-_3$ (mg/l) | $SO_4^{2-}$ (mg/l) | TDS (mg/l) | Electrical Conductivity (µ.S/cm) |
|---|---|---|---|---|---|---|
| April–June 2006 | 50.87 ± 0.016 (60.51 ± 0.006) | 20.89 ± 0.022 (11.13 ± 0.003) | 0.82 ± 0.037 (0.67 ± 0.003) | 4.73 ± 0.038 (2.26 ± 0.006) | 28.87 ± 0.007 (5.65 ± 0.000) | 59.20 ± 0.000 (11.84 ± 0.006) |
| July–Aug 2006 | 57.88 ± 0.003 (58.77 ± 0.003) | 9.96 ± 0.015 (8.91 ± 0.175) | 0.78 ± 0.111 (0.53 ± 0.000) | 1.90 ± 0.069 (2.25 ± 0.126 | 6.73 ± 0.007 (4.80 ± 0.000) | 14.10 ± 0.015 (6.96 ± 0.009) |
| Sept–Oct 2006 | 48.82 ± 0.004 (60.14 ± 0.016) | 14.27 ± 0.006 (6.60 ± 0.013) | 0.76 ± 0.003 (0.63 ± 0.006) | 1.55 ± 0.001 (1.71 ± 0.006) | 7.80 ± 0.000 (5.00 ± 0.000) | 16.55 ± 0.067 (8.35 ± 0.063) |

Data presented as mean ± SEM.

Values in parentheses represent data from Awka City.

N = 8–15 corresponding to episodes of rainfall within the stipulated period.

commonly regarded as determinants of wet deposition chemistry. Anthropogenic emissions can be oxidized to produce carbon, sulfur, and nitrogen compounds in the atmosphere[4] that are balanced stoichiometrically by a net production of $H^+$. Gaseous sulfuric and nitric acids are partially neutralized by gaseous $NH_3$ to form $NH_4NO_3$ and $(NH_4)_2SO_4$, both common components of rainfall influenced by anthropogenic sources. Nitric and sulfuric acids are regarded as the sole contributors of nitrate and sulfate in precipitation[5], and are assumed to be the major sources of acidity in precipitation influenced by combusted hydrocarbons[6]. In our study, both sulfate and nitrate were present, with nitrate being higher, suggesting that considerable $H^+$ is contributed by the dilute mineral acids of nitrogen and sulfur compounds. In another study, precipitation was seasonal, with the largest deposition occurring from October to March, while sulfate had the highest concentration of any anion and accounted for about 40% of the anion total, followed by nitrate[7]. Precipitation also appeared seasonal in the Niger Delta and Awka, characterized by emissions from crude oil–related industries, gas flaring, and sundry anthropogenic activities, especially cottage industries.

Fine nitrate aerosols that dissolved in rainwater are conversion products of $NO_2$, which arises from power plants, other industrial sources, and motor vehicles[8]. Nitrate in drinking water has long been considered a health threat for its ability to induce methemoglobinemia, and this health outcome is the basis of the U.S. Environmental Protection Agency (EPA) maximum contaminant level[9]. Intake of nitrate from drinking water and dietary sources may cause increased exposure to N-nitroso compounds through endogenous nitrosation[10]. In their studies, Coss et al.[11] suggest that long-term exposure to drinking water nitrate at levels below the maximum contaminant level (MCL) of nitrate nitrogen (10 mg/l)[12] is not associated with pancreatic cancer. The average nitrate levels for onset, mid-, and end of rainy season (April–June, July–August, September–October) for 2005 and 2006 in Warri were below the U.S. EPA MCL for nitrate. The average nitrate values from Port Harcourt in 2005 and 2006 for the onset, mid-, and end of rainy season for the 2 years tended to be above the U.S. EPA MCL. High levels of $HNO_3$ concentrations (3.7 µg $HNO_3$ $m^{-3}$, range = 2.7–4.8 µg $HNO_3$ $m^{-3}$) and low levels of $HNO_3$ (0.24 µg $HNO_3$ $m^{-3}$, range = 0.05–0.5 µg $HNO_3$ $m^{-3}$) have been recorded in some places, such as the U.K.[13]. The high level of $NO^-{}_3$ in Port Harcourt, which ranged from 9.96 ± 0.015 to 20.89 ± 0.022 mg/l, is indicative of active precipitation that is of environmental and public health concern.

The work of Niagolova and coworkers[14] explored two hypotheses relating the elevated concentration of nitrogen species in drinking water and the Balkan Endemic Nephropathy (BEN) disease. Their results establish an exposure pathway between anthropogenic activity and drinking water supplies, suggesting that the causative agent for BEN could result from surface contamination. The results from Port Harcourt, an oil-producing city that also has non-oil industrial manufacturing outfits, such as cement, salt, fertilizer, cosmetic and detergent, paints, and rubber processing, seem to implicate an anthropogenic activity. These values were higher than those from Warri. Although the role of nitrate in drinking water as a risk for cancer is controversial, high levels of nitrate in drinking water have been associated with increased mortality[15], and incidences of some cancers and lesions[16]. Spontaneous abortion, ectopic pregnancy[17], malignant lymphomas[18,19], and soft tissues sarcomas[20] have been reported in the Niger Delta region of Nigeria.

Pregnant women who drink nitrate-contaminated water may be at increased risk of having spontaneous abortions[21] or giving birth to infants with congenital malformations, especially of the central nervous system[22]. Turkdogan et al.[23] investigated nitrate and nitrite levels in some traditional foods and drinking water in Van, an endemic upper gastrointestinal (esophageal and gastric) cancer region of eastern Turkey. Their findings suggest that the influence of a traditional diet rich in nitrate and nitrite is significant in the development of upper gastrointestinal cancers in that region.

Sulfate particles in the atmosphere result from the interaction of moisture and $SO_2$, forming acidic solution, and subsequent abstraction of $H^+$ by reactive metals. $SO_2$ is a gaseous compound generated from the burning of sulfur-containing fossil fuel, such as coal and oil, and other sources including power plants, metal smelters (since ores contain metals chemically bound to sulfur needing separation by heat), paper and pulp mills, refineries, and other industrial operations[8].

Hayman et al.[24,25] showed that the largest concentrations of both sulfate and nitrate occur in the period from April to June. This is partly a consequence of the seasonal variation of emissions and of the oxidizing capacity of the atmosphere, as demonstrated by the seasonal variation observed in particulate sulfate concentrations.

Sulfate is one of the least toxic anions of which the WHO does not have any recommended value for drinking water, although catharsis, dehydration, and gastrointestinal irritation have been linked to high sulfate concentrations in drinking water. The WHO therefore suggests urgent action by health authorities when sulfate in drinking water exceeds 500 mg/l (www.who.int/water_sanitation_health/dwq/guidelines/en). So there is near insignificant value of sulfate in our study.

The alkalinity of some water is due only to the bicarbonates of calcium and magnesium, the pH of such water cannot exceed 8.3, and its total alkalinity is identical with its bicarbonate level; the values determined in our study were within acceptable limits. In all the samples, nitrate seems to be the dominant anion. The investigation also revealed (although with minor variation) a direct relationship between TDS and electrical conductivities of all the water samples. We also observed that the TDS and electrical conductivities of rain samples from Port Harcourt were greater than those of Awka (control), followed by that of Warri. In all, the water samples showed levels of TDS below the acceptable range of 500–1500 mg/l[26]. For hardness, two theories have been offered concerning a causative agent for the relationship between death from cardiovascular disease and water hardness. Soft water is more “corrosive” than hard water and promotes the dissolution of cadmium, lead, and other toxic substances from the plumbing system into the drinking water[27]. Another theory is the protective effect of magnesium from the water[28]. Regarding water hardness, there are no distinctly defined levels of what constitutes a hard or soft water supply. A generally accepted classification is that water with less than 75 mg/l of $CaCO_3$ is soft, 75–150 mg/l is moderately hard, and above 150 mg/l of $CaCO_3$ is hard[29].

In our study, all the samples can be said to be soft water, with average samples from Awka and Port Harcourt higher than those of Warri. Taken together, the data of the present study suggest that anthropogenic activities in the Niger Delta impacted on the inorganic ion (nitrate) pollution of the rainwater. Crude oil exploration may not be the cause of acid precipitation since data from Awka, a non-oil exploration city, appeared higher than Warri in some instances and the same in Port Harcourt, both in the Niger Delta. Acid rain, therefore, may be of far-reaching public importance in southern Nigeria. With the nonexistence of public health educators, high infant mortality and poverty tend to accentuate the importance of more epidemiological investigations into the health of the people of the Niger Delta of Nigeria.
